# ﻿Taxonomic notes of the cobweb spider genus *Platnickina* Koçak & Kemal, 2008 (Araneae, Theridiidae, Theridiinae), with descriptions of two new species from southern China

**DOI:** 10.3897/zookeys.1250.158643

**Published:** 2025-08-27

**Authors:** Guolong Huang, Jie Liu, Changhao Hu

**Affiliations:** 1 Arachnid Resource Centre of Hubei Province & Hubei Key Laboratory of Regional Development and Environmental Response, Faculty of Resources and Environmental Science, Hubei University, Wuhan 430062, China Hubei University Wuhan China; 2 Centre for Behavioural Ecology and Evolution, School of Life Sciences, Hubei University, Wuhan 430062, China Hubei University Wuhan China

**Keywords:** Biodiversity, morphology, new combination, review, species group, taxonomy

## Abstract

Based on specimens collected from southern China, two new species of *Platnickina* Koçak & Kemal, 2008 are described: *P. submaculata* Hu & Liu, **sp. nov.** (male, female) and *P. yoshidai* Hu & Liu, **sp. nov.** (male). Two new combinations, transferred from *Theridion* Walckenaer, 1805, are proposed: *P. obscuratum* (Zhu, 1998), **comb. nov.** and *P. xianfengense* (Zhu & Song, 1992), **comb. nov.** Three new species groups within *Platnickina* are established based on morphological characteristics: *antoni* group, *maculata* group, and *sterninotata* group. In addition, we discuss the relationships between *Platnickina* and the genera *Chrosiothes* Simon, 1894, *Coleosoma* O. Pickard-Cambridge, 1882, *Simitidion* Wunderlich, 1992, and *Theridion*.

## ﻿Introduction

The genus *Platnickina* Koçak & Kemal, 2008, a small genus of the family Theridiidae Sundevall, 1833, was originally established as *Keijia* by Yoshida (2001), based on *Keijia maculata* Yoshida, 2001 (= *Platnickina maculata*) as the type species, along with seven species transferred from *Theridion* Walckenaer, 1805. However, the name *Keijia* was preoccupied by a genus in the family Pectocytheridae Hanai, 1957 (Arthropoda, Ostracoda, Podocopida), and was therefore replaced by *Platnickina* for the theridiid genus (Koçak and Kemal 2008) for the theridiid genus. Later, Knoflach (2004) and Wunderlich (2008) each transferred a species from *Theridion* to *Platnickina*, and Gao and Li (2014) described a new species from Yunnan, China. In a recent provisional review of Chinese theridiid species (Zhong et al. 2025), *P. mneon* (Bösenberg & Strand, 1906) was synonymized with *Yunohamella lyrica* (Walckenaer, 1841). To date, this small genus includes 11 extant species distributed worldwide, except for Oceania (Vanuytven 2021; World Spider Catalog 2025). Four of them have been recorded in China: *P. adamsoni* (Berland, 1934), *P. fritilla* Gao & Li, 2014, *P. qionghaiensis* (Zhu, 1998), and *P. sterninotata* (Bösenberg & Strand, 1906) (Zhu 1998; Gao and Li 2014).

This is our sixth taxonomic study on theridiid spiders (Hu 2024; Hu et al. 2024, 2025; Hu and Liu 2025; Zhong et al. 2025). Here, we describe two new *Platnickina* species collected from southern China and transfer two species from *Theridion*, proposing two new combinations.

## ﻿Materials and methods

The specimens examined in this study are deposited in the Centre for Behavioral Ecology and Evolution (**CBEE**), School of Life Sciences, Hubei University, Wuhan. Specimens were examined under OLYMPUS SZX7 and OLYMPUS SZ61 stereomicroscopes. Photographs were taken with an OLYMPUS BX51 microscope and a LEICA M205 C stereo microscope; final multifocal images were produced using Helicon Focus v. 7.7.0. Male palps were examined and photographed after dissection and expanded in warmed lactic acid. Epigynes were dissected from the spiders’ bodies and treated with a warmed 0.1 mg/ml Protease K solution before studying. All morphological measurements were taken using a LEICA M205 C stereomicroscope. Eye diameters were taken at the widest point. Leg measurements are given as total length (femur, patella, tibia, metatarsus, tarsus). All measurements are given in millimeters (mm). The terminology used in the text and figure legends follows Agnarsson et al. (2007).

Abbreviations: A = atrium; ALE = anterior lateral eye; AME = anterior median eye; C = conductor; CD = copulatory duct; CO = copulatory opening; E = embolus; FD = fertilization duct; MA = median apophysis; PLE = posterior lateral eye; PME = posterior median eye; S = spermatheca; ST = subtegulum; T = tegulum; TA = tegular apophysis; I, II, III, IV = legs I to IV.

## ﻿Results

### ﻿Taxonomy

**Family Theridiidae Sundevall, 1833**

**Subfamily Theridiinae Sundevall, 1833**

#### ﻿Genus Platnickina

Taxon classificationAnimaliaAraneaeTheridiidae

Koçak & Kemal, 2008

4DA7040D-3735-51C3-B8AF-094485E01FE7

Keijia Yoshida 2001, type species Keijia maculata Yoshida, 2001 (= Platnickina maculata): 169.
Platnickina Koçak and Kemal 2008 (= Keijia Yoshida, 2001): 3.

##### Diagnosis.

See Yoshida (2001) and Vanuytven (2021).

##### Distribution.

Cosmopolitan excluding Oceania (Vanuytven 2021).

##### Remarks.

Based on the differences in the morphological characteristics of the copulatory organs among *Platnickina* species, we have divided them into three species groups: *antoni*, *maculata*, and *sterninotata* groups.

**The *antoni* group**

**Diagnosis.** The *antoni* group (figs 194–197, 202, 203, 207, 215–218 in Levi 1957) can be distinguished from the *maculata* and *sterninotata* groups by the following combination of characteristics: (1) the ventral part of the median apophysis is anteriorly elongate and pointed; (2) the embolus extends to the dorsal bulb; (3) the diameters of the posterior part of the copulatory ducts are almost as wide as the spermathecae.

**Composition.***Platnickina alabamensis* (Gertsch & Archer, 1942), *P. antoni* (Keyserling, 1884), and *P. punctosparsa* (Emerton, 1882).

**Distribution.** North America.

**The *maculata* group**

**Figure 1.**
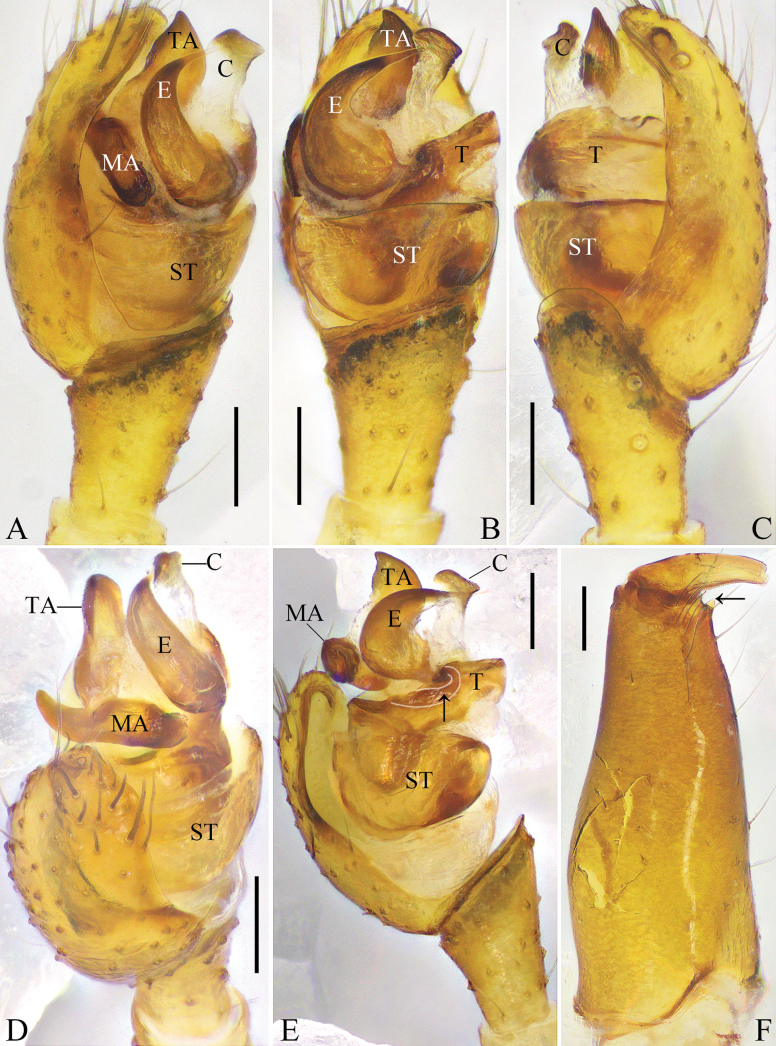
*Platnickina submaculata* Hu & Liu, sp. nov. A. Left male palp, prolateral view; B. Same, ventral view; C. Same, retrolateral view; D. Same, expanded, prolateral view; E. Same, expanded, ventral view, arrow points to the pit on tegulum; F. Right male chelicera, retrolateral view, arrow points to the promarginal tooth. Abbreviations: C = conductor; E = embolus; MA = median apophysis; ST = subtegulum; T = tegulum; TA = tegular apophysis. Scale bars: 0.1 mm.

**Figure 2.**
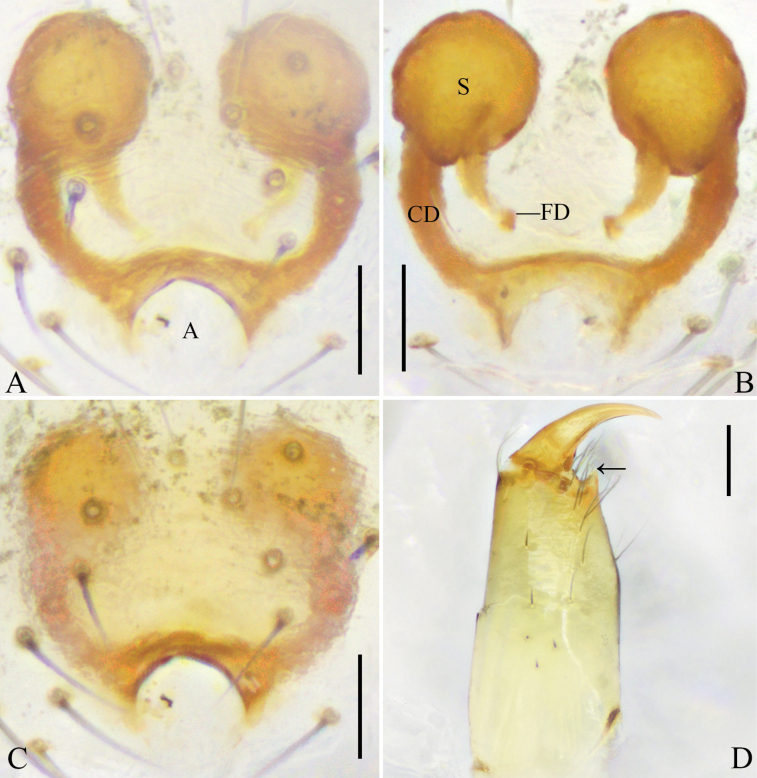
*Platnickina submaculata* Hu & Liu, sp. nov. A. Epigyne, ventral view; B. Vulva, dorsal view; C. Uncleared epigyne, ventral view; D. Right female chelicera, retrolateral view, arrow points to the promarginal tooth. Abbreviations: A = atrium; CD = copulatory duct; FD = fertilization duct; S = spermatheca. Scale bars: 0.05 mm (A–C), 0.1 mm (D).

**Figure 3.**
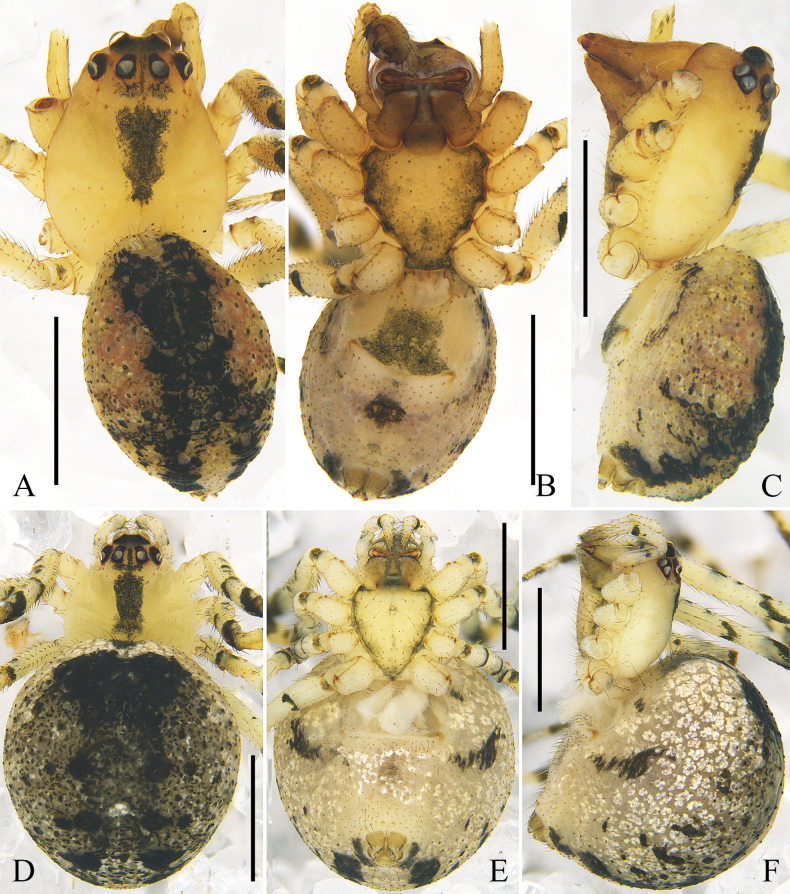
Habitus of *Platnickina submaculata* Hu & Liu, sp. nov. A. Male, dorsal view; B. Male, ventral view; C. Male, lateral view; D. Female, dorsal view; E. Female, ventral view; F. Female, lateral view. Scale bars: 1 mm.

**Figure 4.**
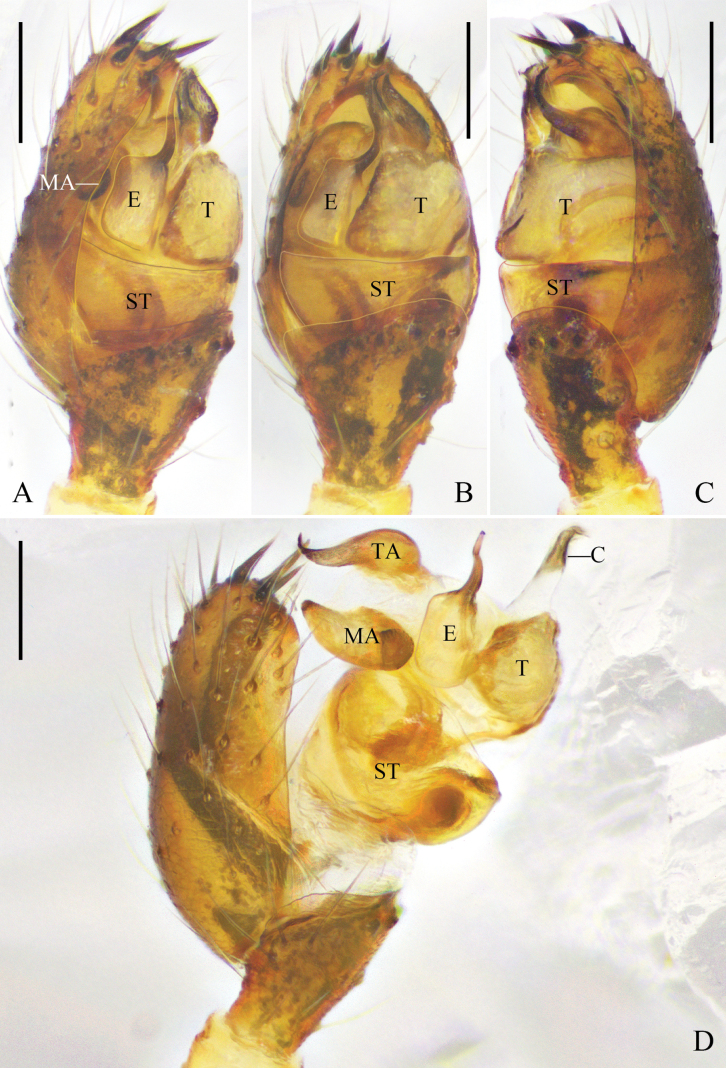
*Platnickina obscuratum* (Zhu, 1998), comb. nov. A. Left male palp, prolateral view; B. Same, ventral view; C. Same, retrolateral view; D. Same, expanded, prolateral view. Abbreviations: C = conductor; E = embolus; MA = median apophysis; ST = subtegulum; T = tegulum; TA = tegular apophysis. Scale bars: 0.1 mm.

**Diagnosis.** The *maculata* group (Figs 1A–E, 2A–C, 3, 4, 6, 7; figs 359–363 in Levy 1998; figs 46–49 in Yoshida 2001; figs 61, 62, 63A, B, 64 in Gao and Li 2014) can be distinguished from the *antoni* and *sterninotata* groups by the following combination of characteristics: (1) the broad tegular apophysis; (2) the thick and cow-horn-shaped embolus (except *Platnickina obscuratum* (Zhu, 1998), comb. nov.); (3) the copulatory ducts straight or slightly curved (except S-shaped in *P. qionghaiensis* (Zhu, 1998)), as long as or shorter than the diameters of spermathecae.

The copulatory organs of the *maculata* group are similar to those of *Simitidion* Wunderlich, 1992 in having a cow-horn-shaped embolus and an epigynal atrium (cf. Figs 1A–E, 2A–C, 3, 4, 6, 7; figs 359–363 in Levy 1998; figs 46–49 in Yoshida 2001; figs 61, 62, 63A, B, 64 in Gao and Li 2014 and figs 595–604 in Wunderlich 1992; figs 1–16 in Knoflach 1996; figs 145–148 in Agnarsson 2007) but can be distinguished from *Simitidion* by: (1) the tegulum ventrally with a pit and the basal part of the embolus with a process; the two structures form a locking mechanism (vs without a locking mechanism between the tegulum and embolus); (2) the median apophysis is separate from the tegular apophysis (vs the median apophysis is fused with the tegular apophysis); (3) the tegular apophysis is wider than the embolus, short and not extending beyond the cymbium, (vs almost as thin as the embolus, long, and extending beyond the cymbium); (4) the copulatory openings without depression (vs with one or two deep depressions).

**Composition.***Platnickina fritilla* Gao & Li, 2014, *P. kijabei* (Berland, 1920), *P. maculata* (Yoshida, 2001), *P. nigropunctata* (Lucas, 1846), *P. obscuratum* (Zhu, 1998), comb. nov., *P. qionghaiensis* (Zhu, 1998), *P. submaculata* Hu & Liu, sp. nov., *P. tincta* (Walckenaer, 1802), and *P. xianfengense* (Zhu & Song, 1992), comb. nov.

**Distribution.** Asia, Europe, North America.

#### ﻿ Platnickina submaculata

Taxon classificationAnimaliaAraneaeTheridiidae

Hu & Liu sp. nov.

908A76B3-72C5-5FFD-AB8C-A6B307934911

https://zoobank.org/280ABC38-22F1-4280-A620-3DD9EE2CA583

Figs 1 , 2 , 3 , 12

##### Type material.

***Holotype*** male (QZMS00885): China – Hubei Province • Enshi Tujia and Miao Autonomous Prefecture, Xuan’en County, Qizimeishan National Nature Reserve, Chunmuying Town, Huoshaobao; 30.0242°N, 109.7565°E; elev. 1919 m; 13 July 2023; Changhao Hu and Mian Wei leg. ***Paratypes*** • 2 females (QZMS05463, 05464): same data as for holotype.

##### Etymology.

The specific name is a combination of the Latin preposition “*sub*” and “*maculata*”, referring to the resemblance of this species with *Platnickina maculata* (Yoshida, 2001); an adjective.

##### Diagnosis.

The male of *Platnickina submaculata* Hu & Liu, sp. nov. is similar to *P. maculata* (Yoshida, 2001) in having a cow-horn-shaped embolus (cf. Fig. 1A–E and figs 48, 49 in Yoshida 2001) but can be distinguished from the latter by: (1) the conductor with a beak-shaped, sclerotized distal part (vs conductor thin, with a pointed tip); (2) the basal part of the embolus wider than the tegulum in ventral view (vs narrower). The male of *P. submaculata* Hu & Liu, sp. nov. is also similar to *P. xianfengense* (Zhu & Song, 1992), comb. nov. in having a cow-horn-shaped embolus and a trapezoidal tegular apophysis (cf. Fig. 1A–E and Fig. 9) but can be distinguished from *P. xianfengense* by: (1) the conductor wide with a beak-shaped distal part (vs with rounded tip); (2) the distal part of the embolus pointing toward the 2:30 o’clock direction in ventral view (vs 1 o’clock direction).

The female of *Platnickina submaculata* Hu & Liu, sp. nov. is similar to *P. fritilla* Gao & Li, 2014 in having simple copulatory ducts (cf. Fig. 2A–C and figs 61C, D, 63A, B in Gao and Li 2014) but can be distinguished from *P. fritilla* by: (1) the atrium almost as large as the spermathecae (vs much smaller than the spermathecae); (2) the copulatory ducts curved (vs straight); (3) the spermathecae widely separated (vs touching). The female of *P. submaculata* Hu & Liu, sp. nov. is also similar to *P. maculata* (Yoshida, 2001) in having a rounded atrium almost as large as the spermathecae (cf. Fig. 2A–C and figs 46, 47 in Yoshida 2001) but can be distinguished by the curved copulatory ducts (vs straight).

##### Description.

**Male**: total length 2.66; carapace length 1.30, width 1.05; opisthosoma length 1.55, width 1.09; eye diameters: AME 0.11, ALE 0.08, PME 0.10, PLE 0.08; eye interdistances: AME–AME 0.15, ALE–AME 0.05, PME–PME 0.10, PLE–PME 0.08, AME–PME 0.10, ALE–PLE 0.00; leg measurements [leg III missing]: I – (2.50, 0.43, 2.38, 1.97, –), II 6.26 (1.98, 0.41, 1.77, 1.59, 0.51), IV 4.32 (1.39, 0.39, 0.93, 1.15, 0.46). Chelicerae with one promarginal tooth, without retromarginal tooth (Fig. 1F).

***Palp*** (Fig. 1A–E): tibia almost 2/3 length of cymbium. Subtegulum bowl-shaped. Tegulum trapezoidal in ventral view. Median apophysis with a rounded ventral part. Tegular apophysis wide, trapezoidal, extending beyond embolus. Conductor membranous, with beak-shaped and sclerotized distal part. Embolus cow-horn-shaped, basal part with a process locking into the tegular pit.

***Colouration*** (Fig. 3A–C): carapace yellow, anterior part light red, with an inverted T-shaped black marking between PMEs; median part of carapace with an inverted triangular black marking. Sternum light orange, with black margins. Chelicerae, endites, and labium light brown. Legs yellow to light orange, with black markings. Dorsal opisthosoma light red, with black flecks, medially with a wide black longitudinal marking branched posteriorly; venter yellowish grey, anterior part with an axe-shaped black marking, posteriorly with a black round marking, laterally with black lines. Spinnerets light brown.

**Female**: total length 2.87; carapace length 1.19, width 1.07; opisthosoma length 1.95, width 1.93; eye diameters: AME 0.12, ALE 0.08, PME 0.09, PLE 0.07; eye interdistances: AME–AME 0.11, ALE–AME 0.04, PME–PME 0.11, PLE–PME 0.08, AME–PME 0.08, ALE–PLE 0.00; leg measurements: I 6.20 (1.98, 0.34, 1.79, 1.59, 0.50), II 5.01 (1.61, 0.34, 1.34, 1.25, 0.47), III 3.44 (1.15, 0.32, 0.64, 0.89, 0.44), IV 3.92 (1.28, 0.36, 0.81, 1.02, 0.45). Leg formula 1243. Chelicerae with one promarginal tooth, without retromarginal tooth (Fig. 2D).

***Epigyne*** (Fig. 2A–C): epigynal field almost as wide as long, posteriorly with a rounded atrium. Copulatory ducts curved, almost as long as diameter of spermathecae. Spermathecae spherical. Fertilization ducts slightly curved, originating from posterior part of spermathecae.

***Colouration*** (Fig. 3D–F): carapace light yellow, medially with a rectangular black marking. Sternum light yellow, with black margins. Dorsal opisthosoma grey, posteriorly with 5 pairs of black spots. Other somatic characters as in male.

##### Distribution.

Known only from type locality (China, Hubei Province) (Fig. 12).

#### ﻿ Platnickina obscuratum

Taxon classificationAnimaliaAraneaeTheridiidae

(Zhu, 1998) comb. nov.

69DA9473-67BC-5580-BF37-812ECB53AAA3

Figs 4 , 5 , 6

Theridion obscuratum Zhu 1998: 167, fig. 105A–D (holotype: female, China, Hubei Province: Enshi Tujia and Miao Autonomous Prefecture, Xuan’en County; 29.90°N, 109.40°E; 26 May 1989; Mingsheng Zhu leg.; deposited in the Museum of Hebei University, Hebei University, not examined); Song et al. 1999: 138, fig. 77G–H (female); Zhang and Jin 2016: 620, figs 1a–b, 2a–e, 3a–d (male, female); Zhou et al. 2024: 3, fig. 3A–D (male).

##### Material examined.

China – Hubei Province • 2 males, 3 females (QZMS02345, 04256, 04257, 04259, 04260); Enshi Tujia and Miao Autonomous Prefecture, Xuan’en County, Qizimeishan National Nature Reserve, Shadaogou Town; 29.6845–29.9238°N, 109.6680–109.7360°E; elev. 685–843 m; 21–24 July 2023; Changhao Hu and Mian Wei leg. – Guangdong Province • 2 females (PWGD2024001, 2024002); Meizhou City, Pingyuan County; 24.8707°N, 115.9361°E; elev. 261 m; 11 July 2024; Chao Liu, Hailun Chen, Jingwei Kang and Yunhe Wang leg. – Guizhou Province • 1 female (PWGZ2024001); Bijie City, Jinsha County; 27.5456°N, 105.9750°E; elev. 826 m; 10 September 2024; Chao Liu, Guolong Huang, Hailun Chen and Yunhe Wang leg. – Hunan Province • 1 female (PWHN2024001); Changsha City, Ningxiang City, Shibasi; 40.0279°N, 112.7215°E; elev. 79 m; 15 August 2024; Chao Liu, Hailun Chen, Jingwei Kang and Yunhe Wang leg.

##### Diagnosis and description.

See Zhang and Jin (2016).

##### Remarks.

**Figure 5.**
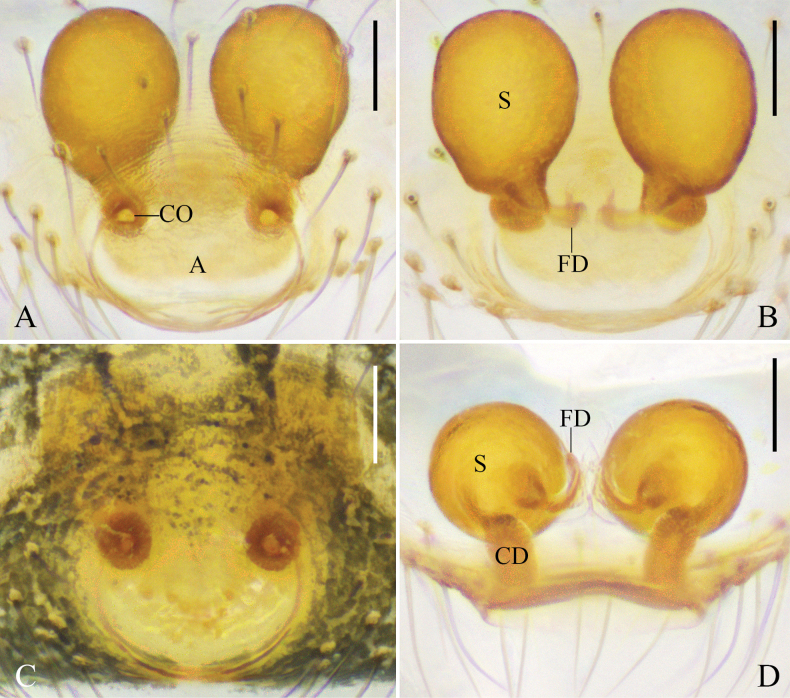
*Platnickina obscuratum* (Zhu, 1998), comb. nov. A. Epigyne ventral view; B. Vulva, dorsal view; C. Uncleared epigyne, ventral view; D. Vulva, posterior view. Abbreviations: A = atrium; CD = copulatory duct; CO = copulatory opening; FD = fertilization duct; S = spermatheca. Scale bars: 0.05 mm.

Although the male of this species possesses an unusual embolus (with a trapezoidal base and thin tip vs cow-horn-shaped in most other *Platnickina* species), the bowl-shaped subtegulum, trapezoidal tegulum, rounded ventral part of the median apophysis, pointed tegular apophysis, and black flecks on the dorsal opisthosoma are consistent with the characteristics of *Platnickina* (Figs 4, 5). In addition, the female has an epigynal atrium, which is diagnostic of the genus (Fig. 5). Therefore, we propose transferring this species to *Platnickina*.

**Figure 6.**
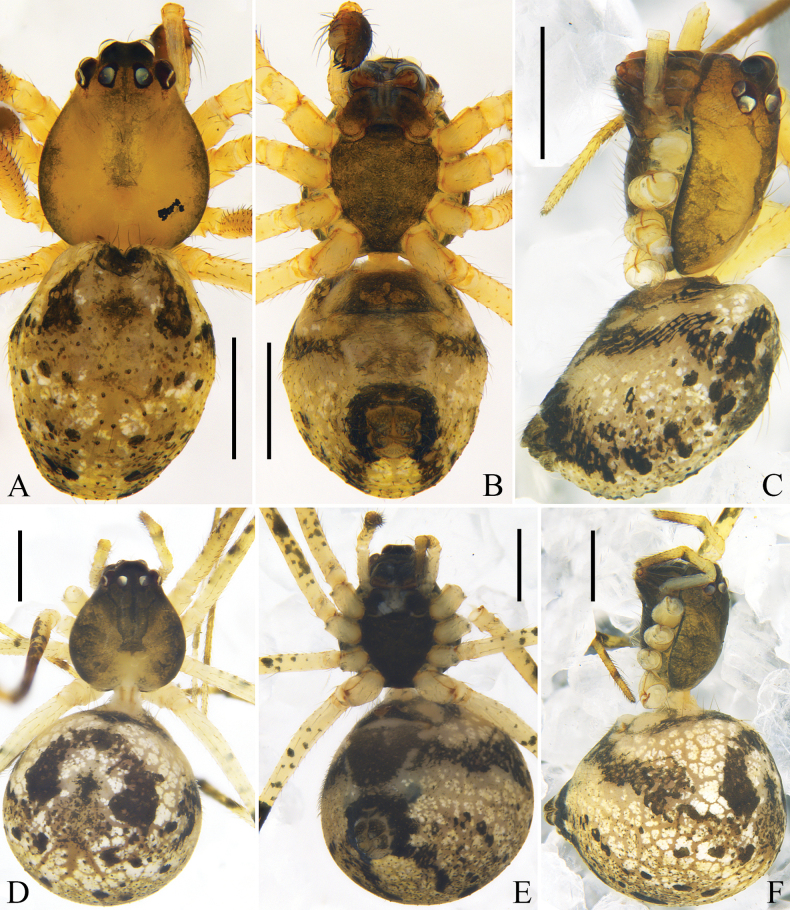
Habitus of *Platnickina obscuratum* (Zhu, 1998), comb. nov. A. Male, dorsal view; B. Male, ventral view; C. Male, lateral view; D. Female, dorsal view; E. Female, ventral view; F. Female, lateral view. Scale bars: 0.5 mm.

##### Distribution.

China (Fujian, Guangdong, Guizhou, Hubei, Hunan).

#### ﻿ Platnickina xianfengense

Taxon classificationAnimaliaAraneaeTheridiidae

(Zhu & Song, 1992) comb. nov.

A883ACF8-FF5B-51AB-886F-CEA1B1FDBD11

Figs 7 , 8 , 9

Theridion xianfengense Zhu and Song 1992: 5, figs I–J (holotype: male, China, Hubei Province: Enshi Tujia and Miao Autonomous Prefecture, Xianfeng County; 31.00°N, 110.05°E; 4 June 1989; deposited in the Museum of Hebei University, Hebei University, not examined); Song and Li 1997: 403, fig. 5A–B (male); Zhu 1998: 154, fig. 95A–E (male, female); Yoshida et al. 2000: 127, figs 19, 20 (male).
Theridion xianfengense Song et al. 1999: 148, fig. 82A, B, K, O (male, female); Yin et al. 2012: 419, fig. 187a–e (male, female); Zhang et al. 2022: 48, fig. 27A–G (male, female).

##### Material examined.

China – Hubei Province • 5 males, 2 females (QZMS02417, 03646–03648, 03651, 04261, 04262); Enshi Tujia and Miao Autonomous Prefecture, Xuan’en County, Qizimeishan National Nature Reserve; 30.0241–30.0764°N, 109.6557–109.7763°E; elev. 645–1777 m; 1–12 July 2023; Changhao Hu and Mian Wei leg. – Fujian Province • 8 males, 6 females (PWFJ2024001–2024014); Zhangzhou City, Zhangpu County; 24.0614°N, 117.6771°E; elev. 80 m; 15 July 2024; Chao Liu, Hailun Chen, Jingwei Kang and Yunhe Wang leg.

##### Diagnosis and description.

See Yin et al. (2012).

##### Remarks.

**Figure 7.**
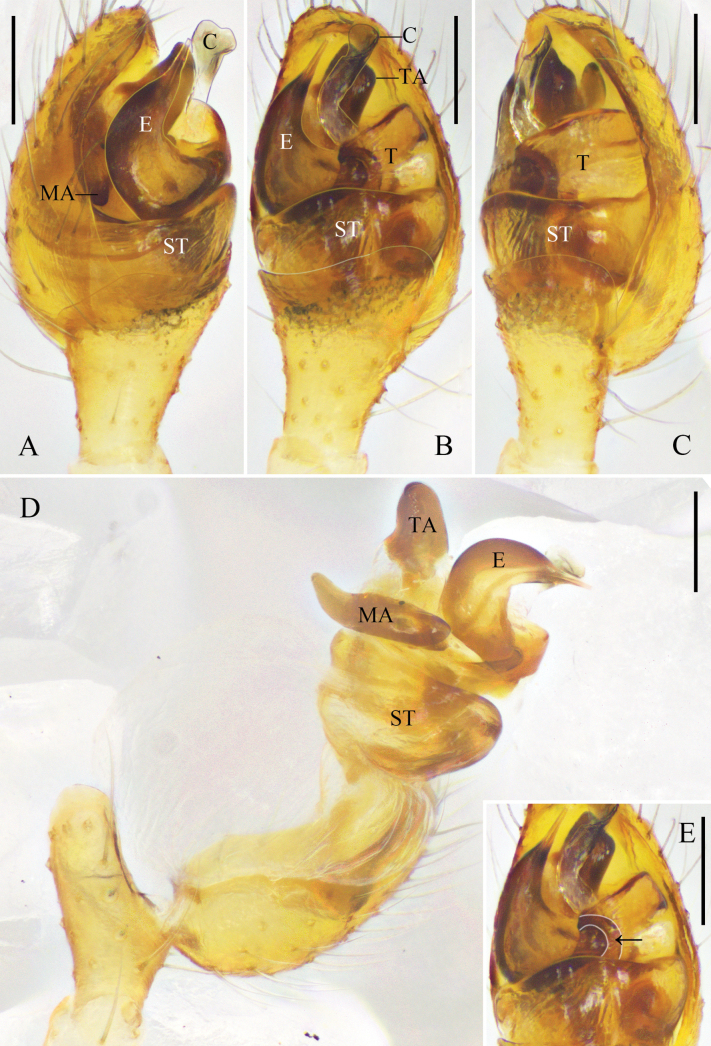
*Platnickina xianfengense* (Zhu & Song, 1992), comb. nov. A. Left male palp, prolateral view; B. Same, ventral view; C. Same, retrolateral view; D. Same, expanded, prolateral view; E. Same, detail of tegulum, arrow points to the pit on the tegulum. Abbreviations: C = conductor; E = embolus; MA = median apophysis; ST = subtegulum; T = tegulum; TA = tegular apophysis. Scale bars: 0.1 mm.

**Figure 8.**
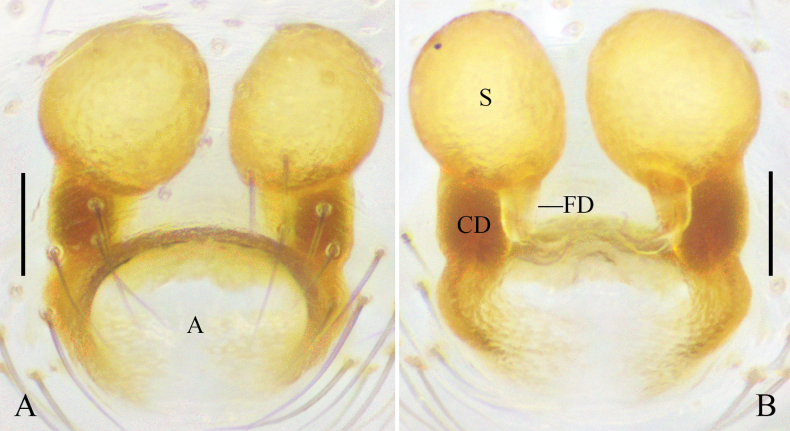
*Platnickina xianfengense* (Zhu & Song, 1992), comb. nov. A. Epigyne, ventral view; B. Vulva, dorsal view. Abbreviations: A = atrium; CD = copulatory duct; FD = fertilization duct; S = spermatheca. Scale bars: 0.05 mm.

**Figure 9.**
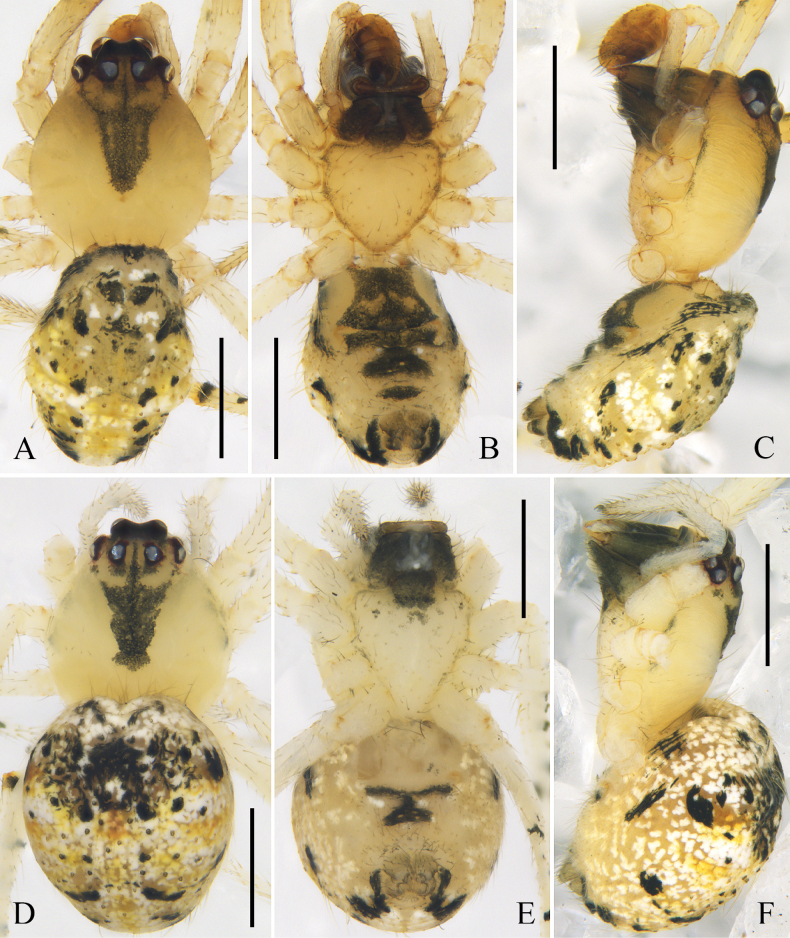
Habitus of *Platnickina xianfengense* (Zhu & Song, 1992), comb. nov. A. Male, dorsal view; B. Male, ventral view; C. Male, lateral view; D. Female, dorsal view; E. Female, ventral view; F. Female, lateral view. Scale bars: 0.5 mm.

This species is similar to *Platnickina maculata*, the type species of the genus *Platnickina*, and shares all the diagnostic characteristics of the genus, including the cow-horn-shaped embolus that forms a locking mechanism with the tegulum, the epigyne with an atrium, and black flecks on the dorsal opisthosoma (Figs 7–9). Therefore, we propose transferring this species to *Platnickina*.

##### Distribution.

China (Chongqing, Fujian, Guangdong, Guizhou, Hainan, Hebei, Hubei, Sichuan, Taiwan).

**The *sterninotata* group**

**Figure 10.**
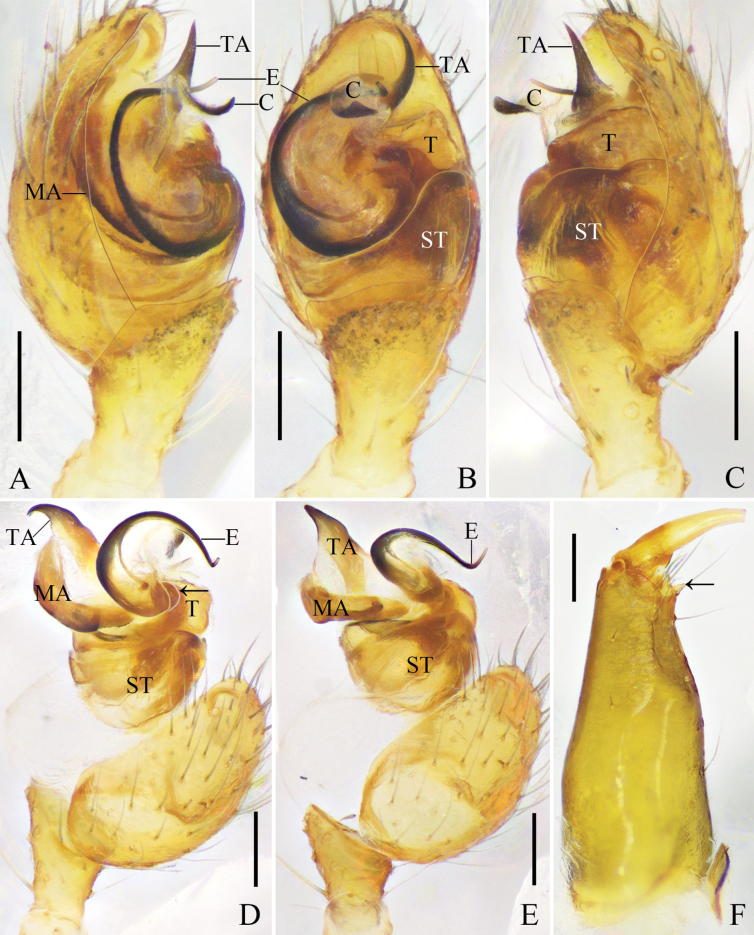
*Platnickina yoshidai* Hu & Liu, sp. nov. A. Left male palp, prolateral view; B. Same, ventral view; C. Same, retrolateral view; D. Same, expanded, prolateral view, arrow points to the pit on tegulum; E. Same; F. Right male chelicera, retrolateral view, arrow points to the promarginal tooth. Abbreviations: C = conductor; E = embolus; MA = median apophysis; ST = subtegulum; T = tegulum; TA = tegular apophysis. Scale bars: 0.1 mm.

**Diagnosis.** The *sterninotata* group (Fig. 10A–E; figs 96B–E, 101B–E in Zhu 1998) can be distinguished from the *antoni* and *maculata* groups by the following combination of characteristics: (1) the embolic base with a membranous retrolateral margin; (2) the atrium with two large copulatory openings; (3) copulatory ducts coiled, obviously longer than the diameters of spermathecae.

Members of the *sterninotata* group are similar to species of *Chrosiothes* Simon, 1894 from eastern Asia in having black flecks on the opisthosoma, the male palp with a membranous embolic basal margin, and the epigyne with a large atrium (cf. Fig. 10A–E; figs 96B–E, 101B–E in Zhu 1998 and fig. 172B–D in Zhu 1998; figs 12–15, 17, 18 in Yoshida et al. 2000) but can be distinguished from the latter by: (1) the median apophysis unbranched (vs branched in Asian *Chrosiothes* species); (2) the atrium with a rounded posterior margin (vs with a triangular posterior margin in Asian *Chrosiothes* species).

Members of the *sterninotata* group are also similar to that of *Coleosoma* O. Pickard-Cambridge, 1882 in having a pointed tegular apophysis (cf. Fig. 10A–E; figs 96B–E, 101B–E in Zhu 1998 and figs 40B, C, F, 41B, C, E, 42B–D in Zhu 1998) but can be distinguished from the latter by: (1) the ventral part of median apophysis rounded (vs pointed); (2) the embolus with a membranous retrolateral base (vs sclerotized totally); (3) the epigyne with an atrium (vs without an atrium, except *Co. floridanum* Banks, 1900).

**Composition.***Platnickina adamsoni* (Berland, 1934), *P. sterninotata* (Bösenberg & Strand, 1906), and *P. yoshidai* Hu & Liu, sp. nov.

**Distribution.** Cosmopolitan, excluding Oceania (World Spider Catalog 2025).

#### ﻿ Platnickina yoshidai

Taxon classificationAnimaliaAraneaeTheridiidae

Hu & Liu sp. nov.

BF47EA16-9240-524A-AD0D-C507E9CC40DE

https://zoobank.org/AE414811-0A1B-4BB4-9B0D-77314130E9A8

Figs 10 , 11 , 12

##### Type material.

***Holotype*** male (PWFJ2024015): China – Fujian Province • Fuzhou City, Jin’an District, Qingyangzuo; 26.0861°N, 119.4012°E; elev. 672 m; 19 July 2024; Chao Liu, Hailun Chen, Jingwei Kang and Yunhe Wang leg.

##### Etymology.

This species is named after the late Japanese arachnologist Dr Hajime Yoshida, who made many important contributions to the taxonomy of theridiid spiders; noun in genitive case.

##### Diagnosis.

The male of *Platnickina yoshidai* Hu & Liu, sp. nov. is similar to *P. adamsoni* (Berland, 1934) in having a pointed and retrolaterally elongate tegular apophysis, and a membranous retrolateral margin of the embolic base (cf. Fig. 10A–E and figs 59–61 in Ono 2011, fig. 5 in Suzuki and Nakama 2021) but can be distinguished from the latter by the shorter embolus, not coiled at the tip (vs longer, tip with horizontal coiling).

##### Description.

**Male**: total length 1.90; carapace length 0.96, width 0.82; opisthosoma length 1.01, width 0.80; eye diameters: AME 0.11, ALE 0.07, PME 0.08, PLE 0.08; eye interdistances: AME–AME 0.10, ALE–AME 0.03, PME–PME 0.06, PLE–PME 0.09, AME–PME 0.08, ALE–PLE 0.00; leg measurements: I 5.08 (1.59, 0.32, 1.52, 1.21, 0.44), II 4.03 (1.25, 0.29, 1.12, 0.95, 0.42), III 2.82 (0.91, 0.23, 0.59, 0.74, 0.35), IV 2.87 (0.90, 0.31, 0.58, 0.72, 0.36). Leg formula 1243. Chelicerae with one promarginal tooth, without retromarginal tooth (Fig. 4F).

***Palp*** (Fig. 10A–E): tibia almost half length of cymbium. Subtegulum with narrow prolateral part and wide retrolateral part. Tegulum small. Median apophysis narrow. Tegular apophysis thin and pointed, elongate retrolaterally. Conductor membranous, with slightly sclerotized distal part pointing ventrally. Embolus curving clockwise, embolic base with broad membranous retrolateral margin, embolic tip pointing ventrally.

**Figure 11.**
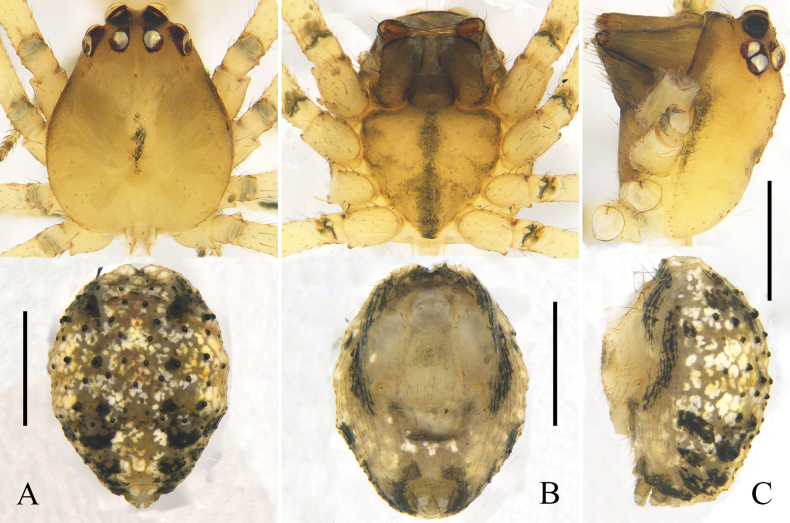
Male habitus of *Platnickina yoshidai* Hu & Liu, sp. nov. A. Dorsal view; B. Ventral view; C. Lateral view. Scale bars: 0.5 mm.

***Colouration*** (Fig. 11): carapace yellow, with black median furrow and lateral margins. Sternum yellow, with black margins and a black longitudinal line. Chelicerae, endites, and labium brown. Legs yellow, with black markings. Opisthosoma yellow; dorsum with white and black spots, and black flecks; venter laterally with black lines. Spinnerets light brown.

**Female.** Unknown.

##### Distribution.

**Figure 12.**
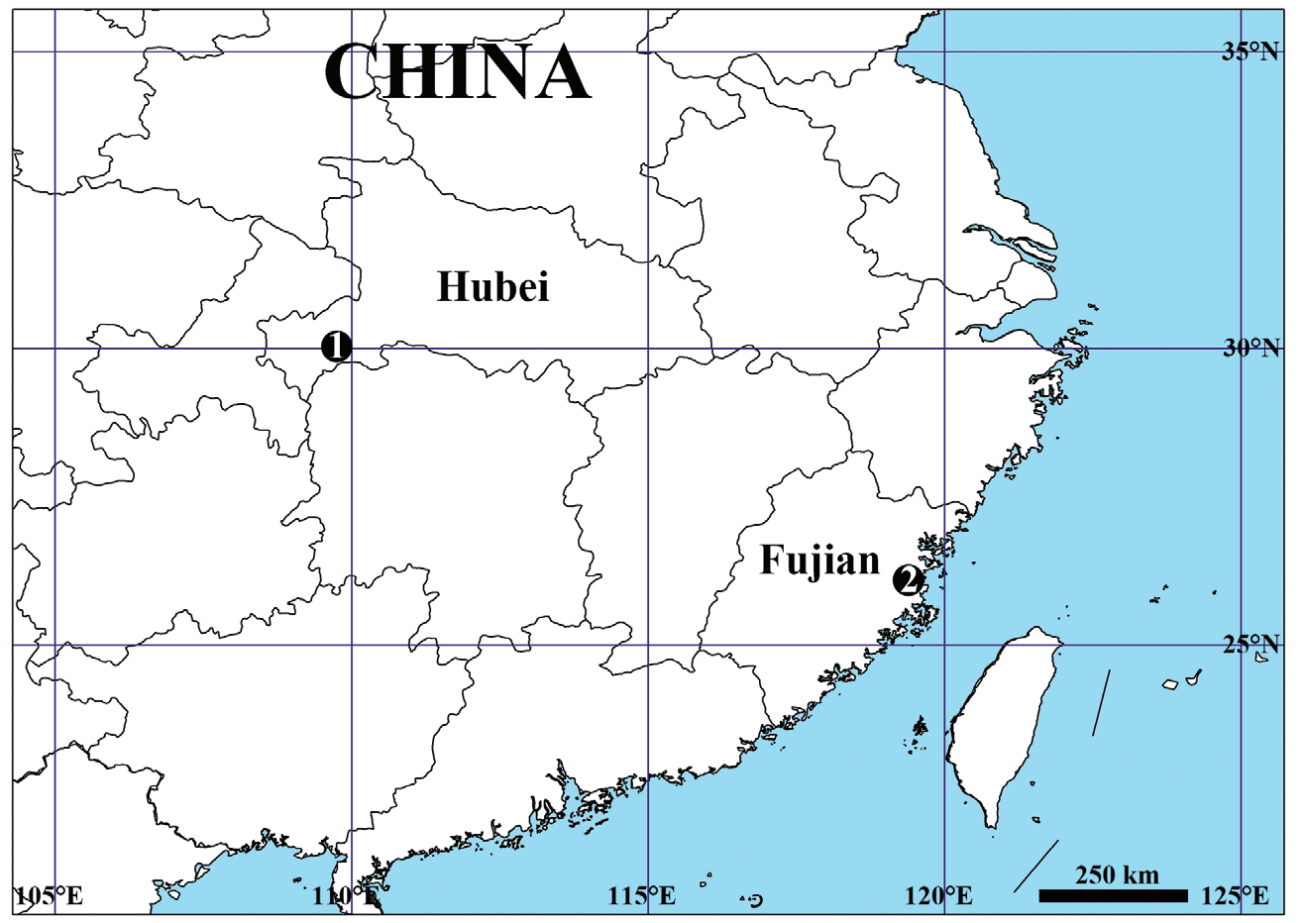
Type localities of the two new *Platnickina* species from southern China. 1 *P. submaculata* Hu & Liu, sp. nov. 2 *P. yoshidai* Hu & Liu, sp. nov.

Known only from type locality (China, Hubei Province) (Fig. 12).

## ﻿Discussion

Yoshida (2001) transferred three North American *Theridion* species (*T. alabamensis*, *T. antoni*, and *T. punctosparsa*) to the genus “*Keijia*” (= *Platnickina*) when he established it but did not provide any justification for these combinations. These three species differ significantly from *P. maculata*, the type species of *Platnickina*, particularly in having a long and pointed median apophysis, a dorsally elongating embolus, and copulatory ducts almost as wide as the spermathecae (Levi 1957). Here, we group these three species within the *antoni* group.

Wunderlich (1992) established *Simitidion*, a genus distributed from the Mediterranean to Central Asia. This genus closely resembles *Platnickina* in the morphology of copulatory organs, e.g. the cow-horn-shaped embolus, and the presence of a broad atrium on the epigyne. However, *Simitidion* lacks the locking mechanism between the tegulum and embolus, a feature present in *Platnickina*. Additionally, the median apophysis in *Simitidion* is fused with the tegular apophysis, whereas these structures are separate in *Platnickina*. These differences suggest that the presence or absence of a tegulum-embolus locking mechanism, as well as the configuration of the median apophysis and tegular apophysis, may represent key diagnostic characteristics at the genus level in theridiid spiders (Agnarsson et al. 2007).

*Platnickina adamsoni* (Berland, 1934) is the most widely distributed species in the genus, recorded from North America, South America, Ghana, South Africa, eastern Asia, and islands in Atlantic, Indian, and Pacific Ocean (World Spider Catalog 2025). In the current paper, we group *P. adamsoni* with *P. sterninotata* (Bösenberg & Strand, 1906) and *P. yoshidai* Hu & Liu, sp. nov. in the *sterninotata* group. This group shares a pointed tegular apophysis with *Coleosoma*. Moreover, both the copulatory organs and somatic characteristics of the *sterninotata* group resemble those of *Chrosiothes* species from eastern Asia [*Ch. fulvus* Yoshida, Tso & Severinghaus, 2000, *Ch. sudabides* (Bösenberg & Strand, 1906), and *Ch. taiwan* Yoshida, Tso & Severinghaus, 2000], e.g. the black flecks on opisthosoma, the membranous basal margin of the embolus, and the large atrium of the epigyne (Zhu 1998; Yoshida et al. 2000; Liu et al. 2016). These two resemblances suggest the possible relationships between the *sterninotata* group and *Coleosoma* and *Chrosiothes*.

*Theridion* is the largest genus in the family Theridiidae, comprising 577 extant species, about 1/5 of the entire family (World Spider Catalog 2025). As the oldest genus in the family, *Theridion* was used as a “dumping ground” for a long time. Different morphological characteristics and polyphyletic relationships indicate that this genus requires taxonomic revision (Agnarsson 2004; Liu et al. 2016). All *Platnickina* species were transferred from *Theridion*. However, many species currently placed in *Theridion*, such as *T. accoense* Levy, 1985 and *T. nagorum* Roberts, 1983, exhibit characteristics consistent with *Platnickina*, particularly the *P. maculata* group (Levy 1998; Saaristo 2010).

## Supplementary Material

XML Treatment for Platnickina

XML Treatment for Platnickina submaculata

XML Treatment for Platnickina obscuratum

XML Treatment for Platnickina xianfengense

XML Treatment for Platnickina yoshidai

## ﻿Additional information

### Use of AI

No use of AI was reported.

### Author ORCIDs

Guolong Huang https://orcid.org/0009-0005-9720-728X

Jie Liu https://orcid.org/0000-0001-7744-9744

Changhao Hu https://orcid.org/0009-0007-5591-3121

### Data availability

All of the data that support the findings of this study are available in the main text.
